# A117 WORK PRODUCTIVITY IMPAIRMENT IN PERSONS WITH INFLAMMATORY BOWEL DISEASES: A SYSTEMATIC REVIEW AND META-ANALYSIS

**DOI:** 10.1093/jcag/gwad061.117

**Published:** 2024-02-14

**Authors:** M Youssef, N Hossein-Javaheri, T Hoxha, C Mallouk, P Tandon

**Affiliations:** Internal Medicine, University of Toronto, Toronto, ON, Canada; Internal Medicine, University at Buffalo, Buffalo, NY; Internal Medicine, University of Toronto, Toronto, ON, Canada; Ottawa University, Ottawa, ON, Canada; University Health Network, Toronto, ON, Canada

## Abstract

**Background:**

Inflammatory bowel diseases (IBD), which include Crohn’s disease (CD) and ulcerative colitis (UC), are chronic disorders that affect approximately 6.8 million people globally. IBD often develops at an early age and can have a significant impact on employment outcomes and work productivity. Individuals with IBD have higher rates of unemployment, sick leave, and work disability compared to the general population. Nevertheless, there remains a paucity of standardized reporting on work productivity impairment and the associated indirect costs in patients with IBD.

**Aims:**

In this systematic review and meta-analysis, we evaluate work-related outcomes and employment data among patients with IBD. This may inform clinical and policy decisions to facilitate workplace accomodations for patients with IBD and improve their work productivity.

**Methods:**

A systematic literature search was conducted in MEDLINE, EMBASE, and the Cochrane library, in addition to Scopus, ProQuest, and clinicaltrials.gov from inception to February 2023. We included full-texts and abstracts of observational and clinical trials that reported work-related outcomes in patients with IBD aged ampersand:003E18 years. We collected work-related data as described by the Work Productivity and Activity Impairment (WPAI) tool, in addition to other outcomes including employment, sick leaves, disability pensions, and indirect costs due to productivity loss. Pooled effect analysis was conducted using a random-effects model for pooled estimates of continuous and proportional data with 95% confidence intervals (CIs). Odds ratios (ORs) and mean differences were used to compare data between patients with active disease vs. remission.

**Results:**

We included 134 studies (96 full-text articles and 38 abstracts). Among all patients with IBD, the pooled estimates were 16.41% [13.89-18.93] for absenteeism, 35.91% [31.11-40.72] for presenteeism, 39.41% [33.93-44.88] for overall productivity loss, and 45.96% [39.46-52.46] for non-work related activity impairment. Indirect costs were estimated at 1,813.9, 3,562.49, and 5,131.09 euros/patient/year for absenteeism, presenteeism, and overall work impairment respectively. For other non-WPAI outcomes, the pooled estimate for employment was 65.6%, 39.5% of patients reported sick days, 21.3% reported disability at work due to IBD directly, 12.3% received disability pensions, and 29.6% had lost jobs due to IBD. Overall, patients with active disease had worse WPAI and employment outcomes than those in remission.

**Conclusions:**

IBD may result in significant work productivity impairment, lower employment, and significant indirect costs due to productivity loss. Larger studies with standardized definitions for work outcomes are needed to better evaluate the impact of IBD on work productivity.

**Funding Agencies:**

Mount Sinai Resident Research Funding

